# Study protocol: a randomised controlled trial investigating the effect of exercise training on peripheral blood gene expression in patients with stable angina

**DOI:** 10.1186/1471-2458-10-620

**Published:** 2010-10-18

**Authors:** Liam Bourke, Garry A Tew, Marta Milo, David C Crossman, John M Saxton, Timothy JA Chico

**Affiliations:** 1 The Centre for Sport and Exercise Science, Faculty of Health and Wellbeing, Collegiate Hall, Collegiate Crescent, Sheffield Hallam University, Sheffield, S10 2BP, UK; 2 School of Allied Health Professions, University of East Anglia, Norwich, NR4 7TJ, UK; 3 NIHR Cardiovascular Biomedical Research Unit, Sheffield Foundation Trust NHS Trust, Herries Road, Sheffield, S5 7AU, UK; 4 Department of Cardiovascular Science, University of Sheffield, Medical School, Beechill Road, Sheffield, S10 2TF, UK

## Abstract

**Background:**

Exercise training has been shown to reduce angina and promote collateral vessel development in patients with coronary artery disease. However, the mechanism whereby exercise exerts these beneficial effects is unclear. There has been increasing interest in the use of whole genome peripheral blood gene expression in a wide range of conditions to attempt to identify both novel mechanisms of disease and transcriptional biomarkers. This protocol describes a study in which we will assess the effect of a structured exercise programme on peripheral blood gene expression in patients with stable angina, and correlate this with changes in angina level, anxiety, depression, and exercise capacity.

**Methods/Design:**

Sixty patients with stable angina will be recruited and randomised 1:1 to exercise training or conventional care. Patients randomised to exercise training will attend an exercise physiology laboratory up to three times weekly for supervised aerobic interval training sessions of one hour in total duration. Patients will undergo assessments of angina, anxiety, depression, and peripheral blood gene expression at baseline, after six and twelve weeks of training, and twelve weeks after formal exercise training ceases.

**Discussion:**

This study will provide comprehensive data on the effect of exercise training on peripheral blood gene expression in patients with angina. By correlating this with improvement in angina status we will identify candidate peripheral blood transcriptional markers predictive of improvements in angina level in response to exercise training.

**Trial Registration:**

Clinicaltrials.gov identifier: NCT01147952

## Background

Regular exercise protects against cardiovascular disease [1] and has beneficial effects on coronary perfusion and ventricular function in patients with established coronary artery disease [2]. However, the exact mechanism whereby exercise training exerts these effects is unclear. Meta-analyses have concluded that exercise based cardiac rehabilitation reduces cardiac mortality in patients with coronary artery disease (prior myocardial infarction, angina, bypass grafting or angioplasty) by 26%-31% [3,4]. Although exercise favourably influences serum lipids and blood pressure, the biological pathway whereby it protects against cardiovascular death remains unclear, though there is substantial evidence that exercise improves myocardial oxygenation. Exercise-induced improvement in myocardial perfusion has been demonstrated in a number of studies [2,5] suggesting exercise training increases absolute myocardial blood flow, rather than by altering myocardial efficiency. Exercise training has also been shown to improve coronary artery endothelial function (even after only 4 weeks training) [6].

Some of the favourable effects of exercise appear to be mediated by improvement in coronary collateral artery recruitment. Exercise training improves collateral vessel function, even in arteries with no coronary stenosis [7]. This is in keeping with epidemiologic evidence that in patients with coronary artery disease, collateral function is better in more active individuals [8]. Since better collateral vessel function is associated with improved survival [9], this is a plausible mechanism for the survival benefit seen with exercise. In keeping with real increases in myocardial perfusion, in one study exercise training has been shown to be better at improving angina than percutaneous coronary intervention [5].

Microarrays enable measurement of expression of almost all human genes in a small clinical sample simultaneously. This powerful technique has been widely applied in cancer, where the relevant tissue is usually easily available either through biopsy, or surgical removal. Application of such techniques is more difficult with heart disease, since the primarily affected organs (the heart itself or its supplying coronary arteries) cannot easily be sampled. However, white blood cells (leukocytes) play a major role in both the development of coronary artery disease [10], in development of collateral vessels [11] and peripheral blood is easily obtainable. Thus, the gene expression of peripheral blood (which reflects gene expression in leukocytes) may be informative in patients with or at risk of cardiovascular disease.

Previous studies have demonstrated the utility of measuring gene expression in peripheral blood or isolated cell types from peripheral blood in patients with cardiovascular disease. These studies found that gene expression in either unsorted peripheral blood [12] or isolated mononuclear cells [13] predicted the presence of coronary artery disease on coronary angiography. Interestingly, gene expression in peripheral unsorted blood accurately reflects expression of the same genes in atherosclerotic arteries [12]. These observations support the validity of peripheral blood as a surrogate of arterial gene expression. This utility exists either because infiltrating leukocytes contribute to arterial gene expression signatures, or pro-atherosclerotic influences alter both arterial and leukocyte gene expression in a similar fashion.

In keeping with the central role of circulating leukocytes (particularly monocytes) in collateral vessel development, microarray profiling of circulating monocytes is able to distinguish between patients with good compared with poor coronary collateral vessels [14,15].

One small previous study assessed the effect of exercise training on peripheral blood gene expression [16]. Five patients with metabolic syndrome exercised for 16 weeks, and significant differences in gene expression were detected in 11 biologic processes. No control group was used but this study does prove that exercise induces measurable changes in gene expression even in a small number of patients.

This study will determine the effect of exercise training on peripheral blood gene expression in patients with stable angina and correlate these changes with improvement in angina status and exercise capacity as a surrogate of myocardial perfusion. In addition, since anxiety and depression are highly prevalent among patients with coronary artery disease and exercise training has been suggested to improve this, [17] we will monitor the effect of our intervention on anxiety and depression.

## Methods/Design

### Study aims

The purpose of this original research is to characterise the effect of exercise training on angina status, anxiety, depression and the peripheral blood transcriptome, in patients with stable angina and coronary artery disease. Gene expression outcomes aim to provide an insight into the mechanism of the beneficial effects of exercise training in coronary artery disease and on collateral blood vessel development which might in turn provide evidence as to the impact upon cardiac morbidity in these patients.

### Study design & setting

This is a prospective randomised control comparison study. After completion of a baseline assessment, participants will be randomly assigned to either a structured exercise or a usual care control group. Patients will be randomised by an independent researcher via code numbers using nQuery software (nQuery Advisor 6.01, Statistical Solutions, USA). The randomisation sequence will not be not disclosed to the researcher responsible for the day-to-day running of the trial until patients have completed baseline assessments. It is not possible to blind participants or investigators to treatment allocation, but data collected will be blinded prior to analysis to eliminate bias. This research will be conducted in the Clinical Research Facility (CRF) at the Northern General Hospital, Sheffield, UK and the Centre for Sport and Exercise Science at Sheffield Hallam University, UK. The researchers involved are experienced clinicians and scientists. Ethical approval was obtained for this study through Bradford Research Ethics Committee.

### Patients

Patients with stable angina will be identified from cardiology clinics at Sheffield Teaching Hospitals NHS Foundation Trust, UK. Written, informed consent will be obtained from each potential volunteer before baseline assessment.

### Inclusion Criteria

1) Class I to III angina pectoris (classified according to the Canadian Cardiovascular Society [CCS]) with documented myocardial ischemia or coronary artery disease on angiography.

2) Ability to read and speak English to a level allowing satisfactory completion of written questionnaires and to understand instruction during the exercise programme.

3) Age 30-80 years of either sex.

### Exclusion Criteria

1) Acute coronary syndromes or recent myocardial infarction (< 2 months).

2) Left main coronary artery stenosis > 25% or high-grade proximal left anterior descending artery stenosis.

3) Known reduced left ventricular function (ejection fraction < 40%).

4) Significant valvular heart disease.

5) Insulin-dependent diabetes mellitus.

6) Occupational, orthopedic, and other conditions that preclude regular exercise.

7) Patients who's ECG prevents interpretation of an exercise test (LBBB, RBBB, pacemaker implantation).

8) Patients already performing greater than 30 min continuous exercise three times weekly (self-reported).

### Exercise intervention

Patients randomised to exercise training will attend an Exercise Science facility based at Sheffield Hallam University. This facility has a range of aerobic exercise equipment, including cycle ergometers, treadmills and cross-trainers. The intervention will comprise sessions of aerobic exercise training for 12 weeks total, up to three times weekly. An interval training regimen will be used, incorporating three minutes of exercise followed by one minute active recovery for 30 minutes total. Heart rate will be monitored throughout; ensuring patients do not exceed 85% of predicted maximum heart rate or 10 b.m^-1 ^below the angina threshold [1]. Individuals who were taking medication that alters maximum heart rate (e.g. beta blockers) will have their exercise intensity monitored using the Borg Ratings of Perceived Exertion [RPE] scale [18]. Exercise intensity in this instance will be set at that point just below the onset of angina. Each exercise session will be preceded by 10-15 minutes of light intensity/gentle mobility exercise and followed by a 10-15 minute period of cool-down activities. If angina is provoked during an interval, exercise will halt until angina subsides, either with rest or GTN treatment. If angina continues longer than 5 min the principal investigator or another clinically qualified investigator will be contacted to assess the patient. Once angina has subsided, blood pressure will be checked, and the intervals will restart at a heart rate 10 b.m^-1 ^below the level which caused the angina symptoms. Subsequent sessions may exceed this level of intensity if the angina threshold is found to have increased (due to exercise training adaptation or alteration of medication).

Hence, each supervised exercise session will last 50-60 minutes in total and patients will be encouraged to attend as many sessions as possible up to three times weekly over the 13 week period. Exercise frequency, intensity, duration and modality, as well as rate of progression, will be logged. After completing the first six weeks, participants will have a week break in training (during which visit 3 takes place), so that acute gene expression changes in response to exercise do not confound the analysis.

### Outcome measures

**Figure 1:**
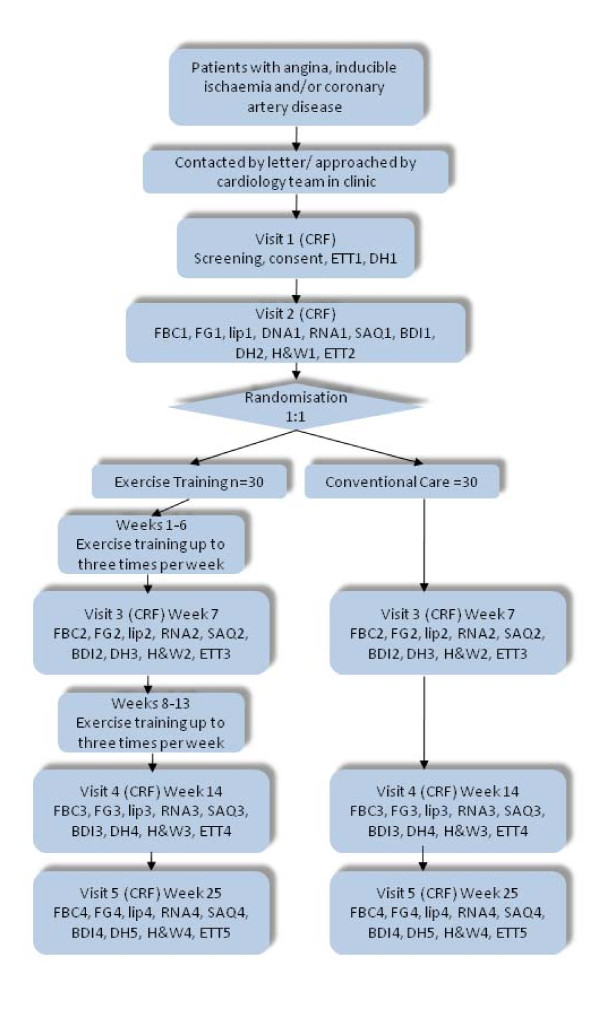
**Flow diagram indicating participant's involvement throughout the course of the study**.

Outcome measures will be performed at CVBRU at baseline, midpoint (week 7), endpoint (week 14), and 6 month follow up. At assessment visits patients will have fasted for at least 12 h. On arrival, the following blood tests will be obtained; full blood count (FBC), fasting glucose (FG), fasting lipids (Lip), and peripheral blood RNA (RNA). The patients will then be offered a light meal. They will complete two questionnaires: The Seattle Angina questionnaire (SAQ), and the Beck Depression/Anxiety Inventory (BDI). The questionnaires are well established, widely used and well validated tools for assessing these outcomes [19-21]. In addition a full drug history will be obtained (DH). Height and weight will also be recorded (H&W). Following completion of these questionnaires, an exercise tolerance test will be performed (ETT) to give an indication or aerobic exercise tolerance and assess angina threshold. RNA samples (stored in a HTA licensed Biorepository) will be extracted and used to bind to Affymetrix Human Genome HU133 microarrays to assess whole genome peripheral blood gene expression. See Figure 1 for study flow diagram.

### Sample size calculation

As our primary outcome measure is gene expression in whole blood, *a priori *sample size calculations are not possible. Based on the pilot study guidelines of [22], we will recruit 30 volunteers for each group, making a total sample size of 60. The results of this pilot will provide information on recruitment, compliance and attrition, and will identify logistical constraints that might limit the number of potential recruits. Variability data for the outcome measures and estimates of the treatment effect could be used to inform design a subsequent larger-scale randomised controlled trial, if post hoc analysis reveals this initial study to be underpowered.

### Statistical analysis

Outcomes will be compared at each assessment point using a mixed-design factorial ANOVA. Where any significant difference existed at baseline between groups, ANCOVA procedures will be implemented, with baseline values used as the covariate [23]. Statistical significance will be set at *p *< 0.05. Bivariate relationships between exercise tolerance and other outcome variables will be evaluated using Pearson's correlation analysis. Data will be analysed using SPSS (SPSS U.K. Ltd, Woking U.K.). The data will be analysed on an intention to treat basis with all analyses including missing data by imputing change across time to be zero. Parametric data will be presented as mean differences of change scores between time points, with 95% confidence intervals and effect sizes (calculated as Cohen's *d *i.e. mean of the treatment effect divided by the standard deviation of the controls). Gene expression data will be analysed using our previously published probabilistic method for analysis of microarray data that improves probe level sensitivity [24-26].

## Competing interests

None of the authors have any competing interests arising from this research.

## Authors' contributions

LB, GT, JS and TC contributed to the design of the study and developed the protocol. LB and TC gained ethical approval for the trial through Bradford Research Ethics Committee. MM and DC contributed to manuscript preparation. All authors approved the final manuscript for submission.

## Pre-publication history

The pre-publication history for this paper can be accessed here:

http://www.biomedcentral.com/1471-2458/10/620/prepub
